# Description of a One-Year Succession of Variants of Interest and Concern of SARS-CoV-2 in Venezuela

**DOI:** 10.3390/v14071378

**Published:** 2022-06-24

**Authors:** Rossana C. Jaspe, Carmen L. Loureiro, Yoneira Sulbaran, Zoila C. Moros, Pierina D’Angelo, Mariana Hidalgo, Lieska Rodríguez, Víctor Alarcón, Marwan Aguilar, Doneyla Sánchez, Jesús Ramírez, Domingo J. Garzaro, José Luis Zambrano, Ferdinando Liprandi, Héctor R. Rangel, Flor H. Pujol

**Affiliations:** 1 Laboratorio de Virología Molecular, Centro de Microbiología y Biología Celular, Instituto Venezolano de Investigaciones Científicas, Caracas 1020, Venezuela; cloureir@gmail.com (C.L.L.); yfsulbara@gmail.com (Y.S.); dgarzaro@gmail.com (D.J.G.); hrangel2006@gmail.com (H.R.R.); 2 Laboratorio de Biología de Virus, Centro de Microbiología y Biología Celular, Instituto Venezolano de Investigaciones Científicas, Caracas 1020, Venezuela; zcmoros@gmail.com (Z.C.M.); jlzr.ivic@gmail.com (J.L.Z.); fliprand@gmail.com (F.L.); 3 Dirección de Diagnóstico y Vigilancia Epidemiológca, Instituto Nacional de Higiene “Rafael Rangel”, Caracas 1053, Venezuela; pierinads@yahoo.com (P.D.); rodriguez.lieska2@gmail.com (L.R.); v.alarcon.fer@gmail.com (V.A.); marwaguilar@gmail.com (M.A.); sdoneyla@gmail.com (D.S.); jesushorn1418@gmail.com (J.R.); 4 Laboratorio de Inmunoparasitología, Centro de Microbiología y Biología Celular, Instituto Venezolano de Investigaciones Científicas, Caracas 1020, Venezuela; mariana.hidalgo.r@gmail.com

**Keywords:** COVID-19, SARS-CoV-2, Variants of Concern, rapid screening, mutations

## Abstract

Some of the lineages of SARS-CoV-2, the new coronavirus responsible for COVID-19, exhibit higher transmissibility or partial resistance to antibody-mediated neutralization and were designated by WHO as Variants of Interests (VOIs) or Concern (VOCs). The aim of this study was to monitor the dissemination of VOIs and VOCs in Venezuela from March 2021 to February 2022. A 614 nt genomic fragment was sequenced for the detection of some relevant mutations of these variants. Their presence was confirmed by complete genome sequencing, with a correlation higher than 99% between both methodologies. After the introduction of the Gamma VOC since the beginning of the year 2021, the variants Alpha VOC and Lambda VOI were detected as early as March 2021, at a very low frequency. In contrast, the Mu VOI, detected in May 2021, was able to circulate throughout the country. After the detection of the Delta VOC in June 2021, it became the predominant circulating variant. With the arrival of the Omicron VOC in December, this variant was able to displace the Delta one in less than one month.

## 1. Introduction

Two and a half years after the detection of the emerging SARS-CoV-2, the disease caused by this coronavirus, COVID-19, has caused more than 500 million reported cases and more than 6 million reported deaths worldwide. This virus belongs to the family *Coronaviridae*. The enveloped virus contains a helicoidal nucleocapsid that surrounds a positive-sense single-stranded genome of approximately 29,900 nt. This genome encodes near the 5′-end for two Open Reading Frames (ORF), coding for 15 non-structural proteins (NSP1-NSp10 and NSP12-NSP16), four genes coding for the four structural proteins (the spike, membrane, envelope, and nucleoprotein), and several genes coding for accessory proteins [1]. The NSPs constitute the replication–translation complex (RTC). This RTC includes an exonuclease, enabling proof-reading capacity and thereby limiting mutational events. However, other factors have led to the emergence of many mutations, such as the great frequency of replication of this virus (due to the extraordinary number of infected persons worldwide), its high rate of recombination, and the probable action of host deaminases on its viral genome, particularly in the spike protein (S) [2,3]. Around 1900 lineages of this virus have been identified [4,5].

WHO classified some of these lineages as Variants of Interest (VOI) or Concern (VOC). VOIs carry mutations that might confer on these viruses a specific phenotypic characteristic, such as higher transmission or immune evasion. VOCs are VOIs for which some of these characteristics have been confirmed. Five VOCs and several VOIs were recognized by WHO [6]. The Alpha VOC (lineage B.1.1.7) emerged in the UK [7], the Beta VOC (lineage B.1.351) in South Africa [8], the Gamma VOC (lineage B.1.1.28.1 or P.1) in Brazil [9], the Delta VOC (lineage B.1.617.2) in India [10], and the last identified Omicron VOC (lineage B.1.529) was first detected in South Africa [11]. In addition to their increased transmission rate, these four later VOCs are also more resistant to the neutralizing activity of antibodies produced during natural infection or vaccination, particularly the Omicron VOC [12,13]. Among the different VOIs, the Lambda VOI (lineage C.37) probably emerged in Peru [14] and the Mu VOI in Colombia (lineage B.1.621) [15]. At present, many of these VOCs and VOIs are not being detected anymore. WHO reduced the list of circulating variants to only two: VOCs Delta and Omicron, with their multiple sublineages [4].

Some key mutations found in the VOIs and VOCs are located in the Receptor-Binding Domain (RBD) of the S protein. Mutation N501Y, present in all except the Delta VOC, confers a higher affinity for the cellular ACE2 (Angiotensin-converting Enzyme 2) viral receptor, and may be related to the increased transmissibility of VOCs carrying this mutation [16]. Mutation E484K, present in the Beta and Gamma VOCs, might reduce the neutralizing activity of antibodies produced by vaccination [17,18]. Mutation L452R of the Delta VOC is associated with an increased transmission potential and reduced susceptibility to protective immunity, both at a humoral and a cellular level [19].

Genomic surveillance was recommended by WHO for monitoring the introduction of SARS-CoV-2 VOCs in each country [20]. However, not all countries possess a robust genome-sequencing capacity, compared to those available in many developed countries. The aim of this study was the monitoring of the succession of VOIs and VOCs for one year in Venezuela, by combining a rapid screening procedure (sequencing of a small genomic fragment) for the initial assignment of variants, with complete genome sequencing for the confirmation of the identity of the variants.

## 2. Materials and Methods

### 2.1. Identification of VOCs or VOIs by Partial Genome Sequencing

**Figure 1:**
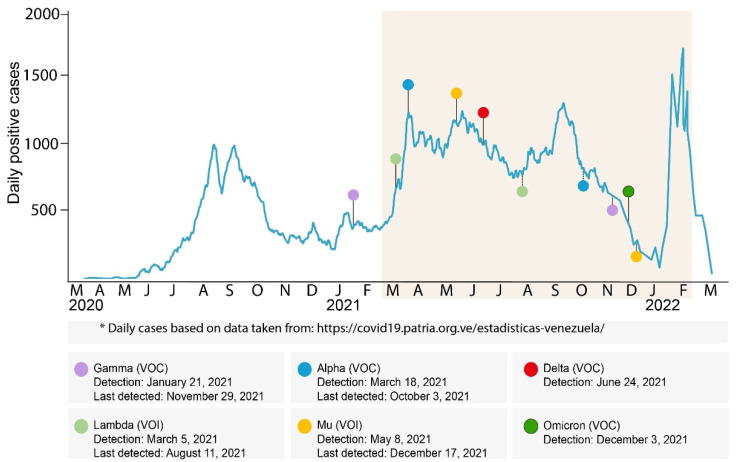
Timeline of cases and detection of VOIs and VOCs in Venezuela. The curve displays the number of COVID-19 cases reported for each day since the first case was detected in Venezuela. The colored shadow corresponds to the period studied. Upper circles represent the date of the first detection for each variant, and lower circles the date of the last detection. The date of the first and the last detection for each variant is based on a complete genome or a partial genome sequence. The date of the first detection of the Gamma VOC was reported previously [21].

This study was approved by the Human Bioethical Committee of IVIC. Samples from nasopharyngeal or nasal swabs and confirmed positive by qRT-PCR during routine COVID-19 diagnosis in Venezuela, from March 2021 to February 2022, were analyzed (Figure 1). The identities of the patients were kept anonymous.

RNA from clinical samples confirmed positive by qRT-PCR was amplified with primers 75L (Artic primer) and 76.8R to generate an amplicon of 614 bp (nt 22.517 to 23.310 of severe acute respiratory syndrome coronavirus 2 (SARS-CoV-2) reference genome), as previously described [21,22]. PCR fragments were sent to Macrogen Sequencing Service (Macrogen, Seoul, Korea). These fragments allowed us to analyze amino acids 345 to 533 of the spike gene, which includes several mutations that allow discrimination of variants (Table 1). The comparison of Ct values between different variants was performed by using the values generated in the same laboratory, using a commercial kit: Novel Coronavirus (2019-nCoV) Nucleic Acid Diagnostic Kit (PCR-Fluorescence Probing) (SANSURE Biotech Inc., Changsha, China).

### 2.2. Complete Genome Sequencing of Selected Isolates

Complete genome sequencing was performed on selected samples by next-generation sequencing, as previously described [21]. Libraries were prepared with a DNA Prep library preparation kit using the Nextera DNA CD Indexes (Illumina, Inc., San Diego, CA, USA), or EasySeq™ RC-PCR SARS-CoV-2 WGS kit (NimaGen BV, Nijmegen, The Netherlands), using SuperScript IV Reverse Transcriptase (Thermo Fisher Scientific, Waltham, MA, USA) with random primers for Nested RT-PCR. Some complete genome sequences were obtained by Sanger sequencing (*n* = 3), as previously described [21]. The libraries were pooled, quantified (Qubit DNA HS, Thermo Scientific), and their quality checked (Bio-Fragment Analyzer, Qsep1-Lite, BiOptic, New Taipei City, Taiwan) before sequencing, which was performed with 10% PhiX control v3, using an iSeq 100 platform and a 300 cycle V2 kit with paired-end sequencing. Viral genome assembly was performed with the Dragen COVID-19 program (Illumina, Inc.) or Genome Detective Virus tool (https://www.genomedetective.com/app/typingtool/cov/, accessed on 30 April 2022). The variant assignment was performed with the Dragen COVID-19 program, Nextclade Web 1.14.1 (https://clades.nextstrain.org/, accessed on 30 April 2022), or Pangolin COVID-19 Lineage Assigner (https://cov-lineages.org/resources/pangolin.html, accessed on 30 April 2022). Nucleotide sequences of complete genomes were deposited into the GISAID database with the accession numbers described in Supplemental Table S1. Illumina DNA Prep Reference Guide (Document # 1000000025416 v09).

### 2.3. Statistical Analysis

Statistical differences between Ct average values for the most frequent variants found in Venezuela were evaluated by the Student *t* test. P values less than 0.05 were considered significant.

## 3. Results

A partial sequencing methodology was developed to allow the screening of a large number of samples for the genomic monitoring of variants. Analysis of 10 amino acid positions allowed for discrimination between the different VOIs and VOCs (Table 1). A total of 7482 samples were analyzed from March 2021 to February 2022. Complete genome analysis was performed in selected samples, according to the following criteria: for confirmation of the detection of a new variant, and to analyze the variant distribution in the different states during the period tested. Complete genome sequences were available for 397 sequences: 11 Alpha, 54 Gamma, 170 Delta, 61 Omicron VOCs, 8 Lambda and 82 Mu VOIs, and 11 samples belonging to other lineages (Table S1). For 390 of these 397 sequences, Sanger sequences obtained by the rapid sequencing method were also available. The correlation of variant assignments between complete genome sequencing and partial genome analysis was more than 99% (Table 2).

Figure 1 shows the number of reported COVID-19 cases since the beginning of the pandemic, the dates of the first detection for each VOC and VOI, and the date of the last detection for some variants. 

**Figure 2:**
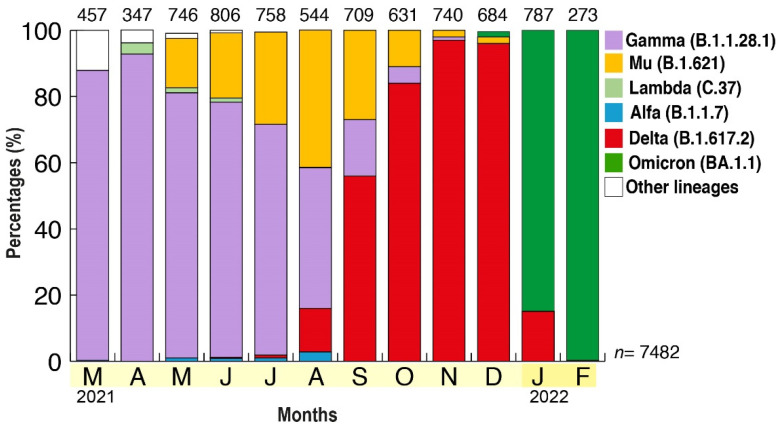
Prevalence of the different VOIs and VOCs in Venezuela from March 2021 to February 2022. The frequency of VOC is shown according to the month of collection of samples in the country. The average frequency was obtained by multiplying the frequency of variants by the population for each state, and then adjusting to 100% if not all the states were tested (see Supplemental Figure S1). The number above each bar refers to the number of samples analyzed each month, *n* = 7482 being the total number of samples analyzed.

The frequency of each VOC and VOI was monitored through time and in each state of the country (Figure 2). A total of 7482 samples were analyzed (Figure 2 and Supplemental Table S2). After the dissemination and predominance of the Gamma VOC (more than 90% in April), other variants began to be detected. Although the Alpha VOC and Lambda VOI were detected since March 2021, their frequency was never more than 3% in a month, each month. The highest frequency for the Alpha VOC was found in August (2.8%, with most of the cases from Zulia State, western Venezuela), and for the Lambda VOI, the highest frequency was found in April (3.4%, and, again, most of the cases were from Zulia State) (Supplemental Figure S1).

The Mu VOI was first detected in May (16%, simultaneously detected in several states, with the highest frequency in Zulia State, Supplemental Figure S1), and increased until August, when 42% of the cases were of the Mu VOC, with an almost equal frequency as the Gamma VOC (Figure 2). The Mu VOI was detected in almost all states of the country during the period from July to September (Supplemental Figure S1).

The Delta VOC was first detected in June 2021, and remained at a low frequency until September, when it reached 50% frequency, and then 96% frequency in November, when it predominated in all the states analyzed (Figure 2 and Supplemental Figure S1). The states where the Delta VOC reached the highest frequency were the North Central states (particularly Distrito Capital and Miranda, Supplemental Figure S1). The Omicron VOC was detected as spread by community transmission in 9 out of the 18 Venezuelan states analyzed during December (Supplemental Figure S1), and predominated in January 2022 in all national territories (Figure 2).

**Figure 3:**
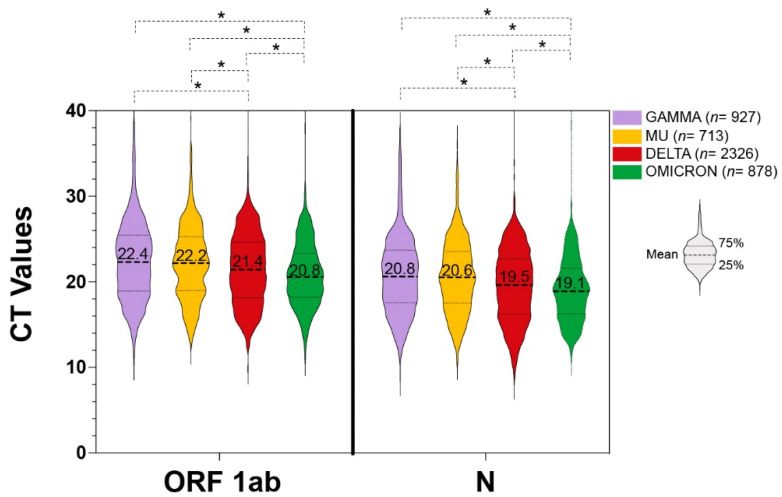
Distribution of the Cycle threshold (Cts) of VOIs and VOCs. The line indicates the average Ct for each variant. VOCs: Gamma (*n* = 928), Delta (*n* = 2322), Omicron (*n* = 878) and VOI Mu (*n* = 714). * Significant differences (*p* < 0.05).

The Cycle threshold (Ct) values were compared between the three VOCs and the one VOI, which were the ones most frequently found in Venezuela: Gamma, Delta, Omicron, and Mu (Figure 3). A significant gradient of decrease in Ct values was observed for the Delta and Omicron VOCs, when compared to any other variant, the Omicron samples being the ones exhibiting the lowest Ct values on average. Between Gamma VOC and Mu VOI, no significant difference was observed. In order to discard any possible confusion because of the different times of circulation of the variants, Ct values were compared for the Delta VOC circulating between September-October 2021 (*n* = 884) and December 2021-February 2022 (*n* = 1617). No significant difference was observed between the two groups (data not shown).

## 4. Discussion

In this study, we developed a simplified methodology for genomic surveillance of the variants of SARS-CoV-2. By analyzing the sequence of 5% of the genome, we were able to detect the different variants that circulated in the country with more than 99% certainty, as determined by complete genome sequencing.

After the introduction and rapid dissemination of the Gamma VOC since the beginning of the year 2021 [21], the next variants detected were the Alpha VOC and the Lambda VOI, in March of the same year. The Alpha VOC was first detected as an isolated case in Miranda State, in March 2021, and then a few cases in some Western states, from May to August. One of these Western States was Apure, which shares a frontier with Colombia. We hypothesize that the Alpha VOC was introduced through international air travel to the Capital region, but also by displacement over land through the frontier with Colombia. In the case of the Lambda VOI, this variant was mainly found in the Andean states of the country, which is consistent with the Peruvian origin of this variant. However, as far as we could detect, neither of these two variants were able to disseminate at a high frequency in any state of the country. Although some degree of resistance to the neutralizing activity of antibodies elicited by some vaccines has been observed for the Alpha VOC, this resistance is lower than the one observed with other VOCs, and even for some of the VOIs [24,25]. This may have hampered the dissemination of Alpha and Lambda variants in Venezuela, as they were probably introduced after the dissemination of the Gamma VOC. It is reasonable to speculate that a significant degree of herd immunity was already acquired in the Venezuelan population after one year of the pandemic, and with the wave due to the Gamma VOC dissemination. In addition, these two variants did not seem able to displace the Gamma one. The low degree of dissemination that occurred in Venezuela for the Lambda VOI is in sharp contrast with the steep increase in this variant observed in Peru, where it became predominant between January and April 2021, despite the co-circulation of other VOCs, such as Alpha and Gamma [26].

The Mu VOI was first detected in May, in the Western states of Venezuela, some of which neighbor Colombia, which is consistent with the possible origin of this variant. The fact that this VOI was first detected at the same time in several states suggests that it could have been introduced earlier into the country. The Mu VOI has been shown to exhibit reduced susceptibility to neutralizing antibodies [24,27,28]. Although never considered a VOC, the Mu VOI was able to displace the Gamma VOC, which was predominating in the whole country, and, after being probably first introduced through the frontier with Colombia (in the Western states, especially Tachira and Zulia, Supplemental Figure S1), it was able to circulate even in the Eastern states of the country. This VOI reached a frequency of circulation similar to the Gamma VOC in August, when the Delta VOC began to replace all the other variants. The pattern of dissemination of the Mu variant is similar to the one observed in Colombia, where this variant also displaced the Gamma VOC [15,29].

It is clear from the many sublineages identified for the Delta VOC (Table 2 and Supplemental Table S1) that multiple introductions occurred of this variant. However, in contrast to the Gamma and Mu variants, for which a frequent flow of introduction probably occurred by ground from the Eastern and Western frontiers of Venezuela, respectively, the Delta VOC reached its highest frequency first in the Metropolitan region, suggesting that this variant was frequently introduced by international travelers.

A limitation of this study is that partial sequencing does not allow for differentiation among the many sublineages of the variants. Several sublineages of the Gamma VOC and the Mu VOI have already been described but none of them were found among the complete genome sequences obtained, while 17 different sublineages could be identified for the Delta VOC. Because of the relatively small number of complete genome sequences available, we cannot discount that some of the sublineages of the Gamma and Mu variants might have been introduced in Venezuela. In addition, these sublineages may have been limited in their dissemination by the introduction of the Delta VOC.

After its first detection on December 8, 2021, in international travelers returning to Venezuela [30], the Omicron VOC was soon detected as spreading through community transmission in 9 out of the 18 Venezuelan states analyzed that same month (Supplemental Figure S1). The first community-acquired Omicron sample was detected on December 3 (data not shown). While it took two months for the Delta VOC to displace the other circulating variants, it took less than one month for the Omicron VOC variant to displace the Delta one and to predominate in January 2022 (Figure 1). In the case of the Omicron VOC, our simplified sequencing protocol allows us to discriminate among the sublineages of the Omicron VOC BA.1 to BA.5. No evidence of the circulation of sublineages other than BA.1 and BA.1.1 was found in Venezuela until February 2022. After the epidemic wave of the Omicron BA.1/BA.1.1 that was observed in January/February, an abrupt decrease in the cases was observed (Figure 1). Assuming that the Omicron BA.2 did not circulate in the country until at least February 2022, a moderate new epidemic wave could be expected if one of these other lineages of Omicron arrives, as occurred in many countries in Europe and the USA.

The average Ct values were inversely correlated with the emergence of each of the variants which were able to disseminate and then replace the previous variant circulating. The observed reduction in average Ct values might be related to the higher transmissibility of the variants that were subsequently predominant in Venezuela [24]. However, from the obtained data we can only hypothesize this possibility, because there are several limitations hampering the comparison of Ct values in this study: the lack of stratification by the presence or not of symptoms, by the specific day of symptom onset, the lack of longitudinal data to measure the peak viral load, and the use of Ct as a surrogate measure of viral load without standard controls. However, since the analysis was performed in a relatively high number of samples, we found it relevant to show the observed reduction in Ct values during the succession of these variants in Venezuela.

Another factor that may have shaped the intensity of the epidemic and the dissemination of the variants is the vaccine coverage during the epidemic in Venezuela. According to the data available in PAHO [31], Venezuela effectively began vaccination at the beginning of August 2021, and reached about 50% of two-dose vaccine coverage by the beginning of 2022, with 75% of the population having received at least one dose at this time. The most frequently used vaccines in Venezuela were the Beijing CNBG—BBIBP-Corv, followed by Gamaleya Sputnik. Since vaccination started relatively late in the country, it might not have impacted the dissemination of the variants in Venezuela, except perhaps the Omicron VOC, known to be the most resistant to the immune protection mediated by vaccination [13].

The successive waves and replacement of variants in Venezuela are also consistent with the relative fitness evaluated for them [32]. According to this review, the relative average fitness of the Omicron BA.1 has been estimated around 60% higher than that of the Delta VOC, which in turn is around 50% higher than that of the Gamma VOC. The Mu VOI displayed on average a slightly higher fitness compared to the Gamma VOC, which might explain that this VOI was able to circulate in the whole country despite the presence of the Gamma VOC. In contrast, the founder effect of the Gamma VOC did not allow the dissemination of the Alpha VOC or the Lambda VOI, which exhibit lower or similar fitness, respectively, compared to the Gamma one [32].

## 5. Conclusions

By using a partial sequencing strategy and then confirmation with complete genome sequencing, we were able to detect and monitor the introduction and variable dissemination of VOCs and VOIs in Venezuela. The epidemiological behavior of the variants detected was similar to the one described in other countries, particularly in Latin America [33].

## Figures and Tables

**Table 1:** Mutations analyzed in Sanger sequencing for variant assignment.

Variant	Alpha	Beta	Gamma	Delta	Omicron *	Lambda	Mu
D405					N (BA.2/3/4/5)		
R408					S (BA.2/5)		
K417		N	T	N (AY.1) **	N		
N440					K		
G446					S (BA.1)		
L452				R	R (BA.4/5)	Q	
T478				K	K		
E484		K	K		A		K
F490						S	
N501	Y	Y	Y		Y		Y

Mutation information is available at [23]. * Omicron VOC exhibits other mutations in this region, but they were not necessary for the proper assignment of this variant. Mutation D405N is present in Omicron sublineages BA.2 to BA.5 but not in BA.1, and R408S is only present in BA.2. and BA.5. ** Mutation K417N is present in only one sublineage of VOC Delta: AY.1.

**Table 2:** Correlation between variant assignment by Sanger and NGS sequencing.

Variant	NGS	Sanger
Alpha	11	11
Gamma	54	52 *
Delta	163 (several sublineages) **	163
Omicron	61 ***	61
Lambda	8	8
Mu	82	82
Other lineages	11	11
Correlation	388/390 (99.5%)	

* In one sample, the mutated nucleotide which codes for the K417N mutation was misleadingly read in the Sanger sequencing, suggesting a Mu variant instead of a Gamma one. The other sample exhibited a mixed pattern P1/Delta due to mixed infection or contamination in the Sanger sequence. ** The sublineages found for Delta VOC were, in addition to B.1.617.2: AY.3, AY.5, AY.20, AY.24, AY.25, AY.25.1, AY.26, AY.33, AY.42, AY.43, AY.44, AY.100, AY.103, AY.122, AY.122.4, and AY.127, with AY.122 being the most abundant (Supplemental Table S1). *** Omicron lineages BA.1 and BA.1.1 (Supplemental Table S1).

## Data Availability

Not applicable.

## Supplementary Materials

The following supporting information can be downloaded at: https://www.mdpi.com/article/10.3390/v14071378/s1, Figure S1: Frequency of variants according to state and month; Table S1: GISAD accession number for the complete genome generated in this study, with the lineage defined according to the Pangolin classification [4]; Table S2: Number of partial genomic sequences available per month in each state of Venezuela.
