# Supplementary figures and images for: Description of a One-Year Succession of Variants of Interest and Concern of SARS-CoV-2 in Venezuela
Source: Viruses. 2022 Jun 24;14(7):1378. doi: 10.3390/v14071378 (PMC9317613; doi:10.3390/v14071378)

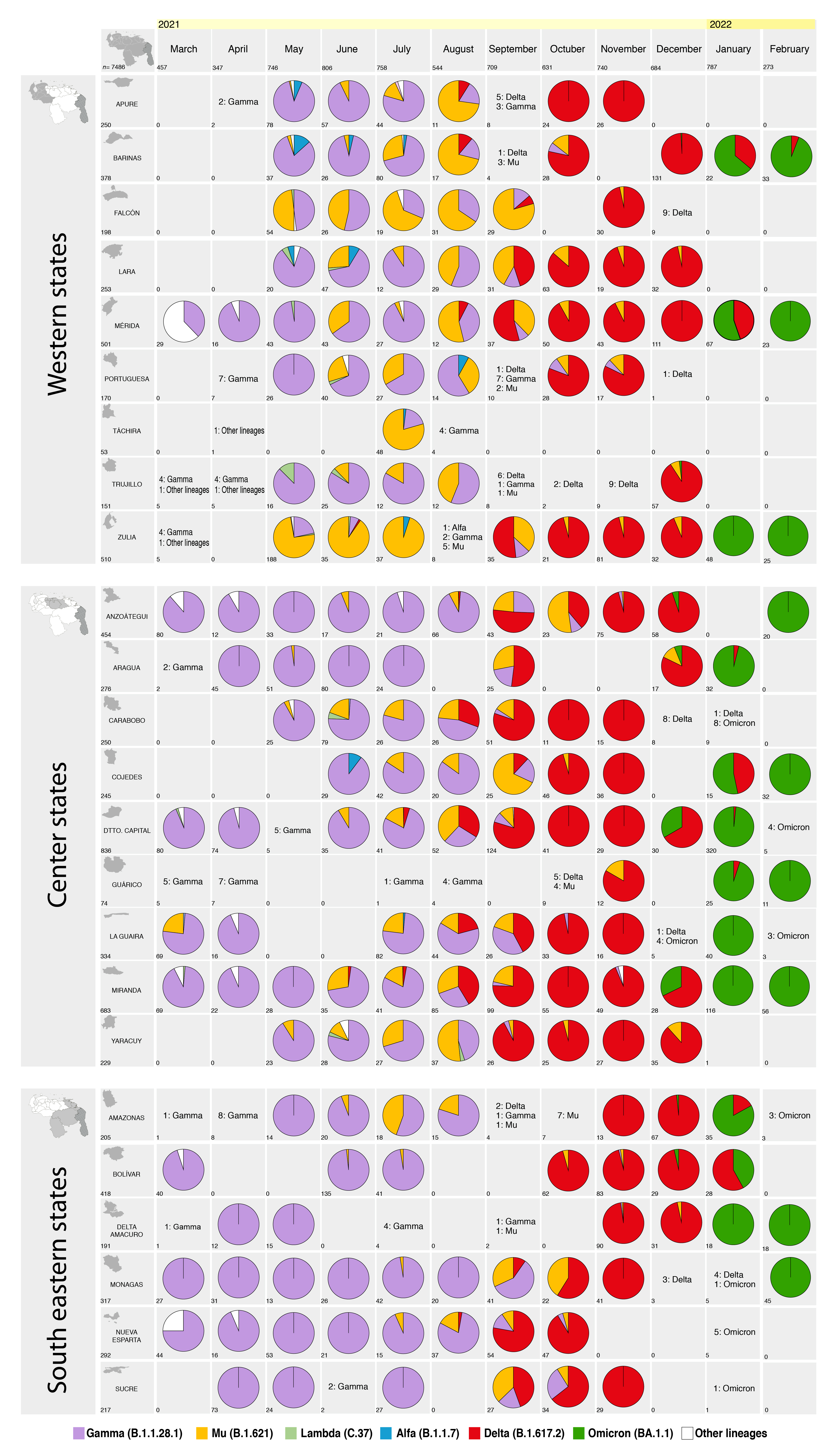

Supplement: Supplementary file 1 [file viruses-14-01378-s001.zip › Figure S1.jpg]
